# A Telehealth Diabetes Intervention for Rural Populations: Protocol for a Randomized Controlled Trial

**DOI:** 10.2196/34255

**Published:** 2022-06-14

**Authors:** Michelle L Litchman, Bethany M Kwan, Linda Zittleman, Juliana Simonetti, Eli Iacob, Kristen Curcija, Julie Neuberger, Gwen Latendress, Tamara K Oser

**Affiliations:** 1 College of Nursing University of Utah Salt Lake City, UT United States; 2 Utah Diabetes and Endocrinology Center University of Utah Salt Lake City, UT United States; 3 Department of Family Medicine University of Colorado School of Medicine Aurora, CO United States; 4 Department of Internal Medicine University of Utah Salt Lake City, UT United States

**Keywords:** diabetes, rural, telehealth, implementation science, community-based participatory research, Spanish, implementation, community, participatory, protocol, randomized controlled trial, intervention, adapt, framework

## Abstract

**Background:**

Diabetes self-management education and support (DSMES) is a crucial component of diabetes care associated with improved clinical, psychosocial, and behavioral outcomes. The American Association of Diabetes Care and Education Specialists, the American Diabetes Association, and the American Academy of Family Physicians all recommend DSMES yet accessing linguistically and culturally appropriate DSMES is challenging in rural areas. The Diabetes One-Day (D1D) program is an established DSMES group intervention that has not been adapted or evaluated in rural communities.

**Objective:**

The specific aims of this paper are (1) to adapt the existing D1D program for use in rural communities, called rural D1D (R-D1D); and (2) to conduct a patient-level randomized controlled trial to examine the effects of R-D1D and standard patient education, guided by the Reach, Effectiveness, Adoption, Implementation, and Maintenance framework.

**Methods:**

This is a protocol for a pilot type II hybrid implementation-effectiveness trial of a culturally adapted virtual DSMES program for rural populations, R-D1D. We will use Boot Camp Translation, a process grounded in the principles of community-based participatory research, to adapt an existing DSMES program for rural populations, in both English and Spanish. Participants at 2 rural primary care clinics (4 cohorts of N=16 plus care partners, 2 in English and 2 in Spanish) will be randomized to the intervention or standard education control. The evaluation is guided by the Reach, Effectiveness, Adoption, Implementation, and Maintenance framework. Patient-level effectiveness outcomes (hemoglobin A_1c_, diabetes distress, and diabetes self-care behaviors) will be assessed using patient-reported outcomes measures and a home A_1c_ test kit. Practice-level and patient-level acceptability and feasibility will be assessed using surveys and interviews.

**Results:**

This study is supported by the National Institute of Nursing. The study procedures were approved, and the adaptation processes have been completed. Recruitment and enrollment started in July 2021.

**Conclusions:**

To our knowledge, this will be the first study to evaluate both effectiveness and implementation outcomes for virtually delivered DSMES, culturally adapted for rural populations. This research has implications for delivery to other rural locations where access to specialty diabetes care is limited.

**Trial Registration:**

ClinicalTrials.gov NCT04600622; https://clinicaltrials.gov/ct2/show/NCT04600622

**International Registered Report Identifier (IRRID):**

DERR1-10.2196/34255

## Introduction

### Diabetes and Rural Populations

Diabetes mellitus is a chronic, progressive disease affecting 30.3 million people in the United States [1]. In addition to being the 7th leading cause of death in the United States, diabetes contributes to serious microvascular and macrovascular complications [1]. Diabetes self-management is the cornerstone to avoiding or delaying diabetes complications, and ongoing self-management is necessary. Self-management behaviors involve an often-challenging daily diet, medication, exercise, and glucose monitoring regimen, among others, and is complicated by social determinants of health (eg, transportation and access to high-quality care). Diabetes self-management education and support (DSMES) is a crucial component of diabetes care that provides the foundation necessary to self-manage diabetes. The American Association of Diabetes Care and Education Specialists, the American Diabetes Association, and the American Academy of Family Physicians all recommend DSMES [2]. DSMES can improve outcomes in both type 1 (T1D) and type 2 diabetes (T2D), including improving hemoglobin A_1c_, quality of life, diabetes distress, and healthy coping [3-6]. However, there is variation in how DSMES interventions are structured, in terms of both content and delivery, and as such, some DSMES programs may be more acceptable, feasible, and effective than others [7].

Approximately 50% of adults with T1D [8] and 90% of adults with T2D in the United States are managed in primary care settings and not in endocrinology specialty practice offices [9], and therefore often lack access to services such as DSMES. Strikingly, 75% of counties in the United States have no endocrinologists at all, while primary care can be found in 96% of US counties [10]. Especially in rural areas, many people with diabetes receive their diabetes care at primary care practices and may have to travel long distances to access DSMES when not available in their medical home. When DSMES is available, there can be a lack of fit between the curriculum, materials, or mode of delivery and the resources of rural primary care practices and communities, including a lack of staff and lack of information tailored to the resources and culture in rural communities. There is a clear need for DSMES interventions, feasible for delivery in rural settings, that can address the unique social determinants of health and cultural adaptations appropriate for people with diabetes living in rural communities.

The 2017 National Standards for DSMES recommend a multidisciplinary team delivering DSMES, a curriculum reflecting current evidence and practice guidelines, individualized plans, and sources for ongoing support [11]. Personalizing care via DSMES is critical because it allows individuals to identify how to incorporate self-management into their own daily life [12,13]. While individual DSMES may be better suited for personalized approaches to care, group sessions can be more cost-effective than individual sessions [14] and offer opportunities for peer support. However, when sessions are held over several weeks, it can be challenging for individuals to complete leading to attrition [15]. A systematic review of 118 unique DSMES interventions found that patients who receive group and individual DSMES sessions, from multidisciplinary teams, are the most effective at reducing A_1c_ [15]. Interventions that address the need for group and personalized care can reduce disparities in rural populations. This study will address current gaps in rural diabetes care research and a widely recognized need to enhance access to DSMES among rural populations.

National data indicate that diabetes prevalence is higher in rural areas. Rural-dwelling adults are 17% more likely to have diabetes than their urban counterparts nationally [16]. For instance, the rates of diabetes in rural eastern Colorado average 12.3% compared to the state average of 7.3% [17]. Rural populations have limited access to DSMES, and health care access is more limited in rural areas, impeded with long travel distances, as well as higher rates of poverty than those individuals in urban settings [14]. These challenges contribute to a greater risk of suboptimal diabetes management and higher rates of diabetes-related complications [18]. The Diabetes One-Day (D1D) intervention is a condensed, one-day group DSMES session delivered via telehealth by a multidisciplinary team at a diabetes and endocrinology center. Patients are encouraged to invite a care partner (family member or friend) to attend D1D given that diabetes is managed in a social context. D1D was designed to address DSMES access challenges seen with multiple-day DSMES interventions. Evidence suggests that the D1D intervention is effective for improving diabetes self-management outcomes [19,20] and is a promising intervention to address specialty care access challenges seen in rural communities. Further consideration of cultural and social adaptations to D1D (ie, a Rural D1D [R-D1D] program) is needed to ensure fit for rural communities, especially those where English and Spanish are the primary languages spoken. We describe our protocol for adapting D1D for rural primary care and implementing, evaluating, and refining the resulting R-D1D intervention.

### Intervention Logic Model

**Figure 1:**
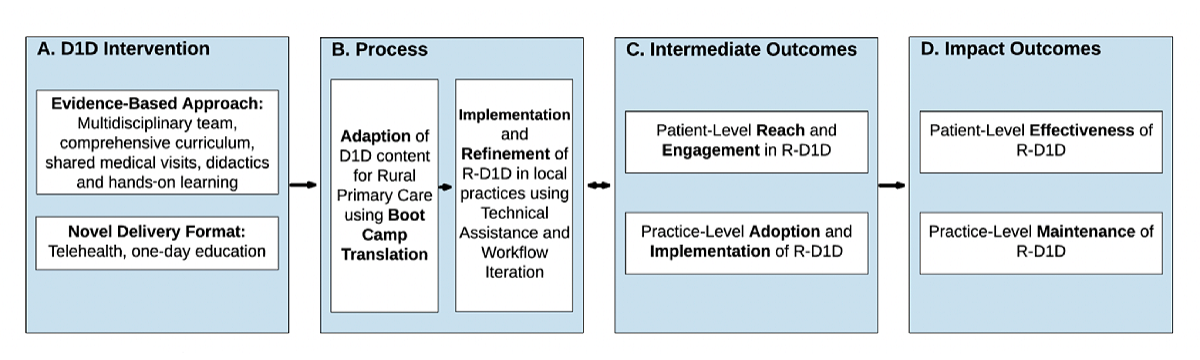
Intervention logic model. D1D: Diabetes One-Day; R-D1D: Rural Diabetes One-Day.

The logic model for this project (Figure 1) depicts the process by which the D1D intervention (Box A) is adapted, implemented, and refined for rural settings (R-D1D, Box B), to promote the successful adoption and implementation by rural practices and reach to patients in the target audience (Box C). Once adopted and implemented, a future study will assess effectiveness for patient-reported and clinical outcomes at the patient level and maintenance at the practice level (Box D). That is, to achieve impact on patient-level outcomes and to be sustainable for practices, the intervention must be acceptable, appropriate, and feasible for delivery in rural primary care, be implemented well, and reach target populations. The intermediate outcomes and impact outcomes align with the Reach, Effectiveness, Adoption, Implementation, and Maintenance (RE-AIM) framework, a well-established framework for program planning and evaluation [21].

### Aims and Hypothesis

The coprimary aim of this study is to adapt the existing D1D program for use in rural communities (R-D1D). Boot Camp Translation (BCT) [22,23] will be used to partner with diverse community members, including members from Spanish-speaking communities, to adapt diabetes self-management language and tools and delivery of D1D into a culturally relevant R-D1D for rural primary care practices, patients, and care partners.

The secondary coprimary aim is to conduct a patient-level pilot randomized controlled trial (RCT) to examine the effects of R-D1D (intervention group) vs standard patient education (attention control group), guided by the RE-AIM framework. We will implement and refine the R-D1D intervention, evaluate implementation at the practice level, and evaluate reach and effectiveness at the patient level.

## Methods

### Design

This study will use the BCT method to adapt the medical information, guidelines, and materials in the current D1D intervention for use with English-speaking and Spanish-speaking people living in rural eastern Colorado (R-D1D). Then, using a 1:1 type 2 hybrid trial, we will conduct a pilot test in 64 total patients from 2 practices to compare R-D1D to a standard education control. Acceptability and feasibility of R-D1D will be assessed at the patient and practice levels.

### Setting

This project will take place in the High Plains Research Network, a practice-based research network of 53 primary care practices, hospitals, and communities in a 16-county region of rural eastern Colorado. Two High Plains Research Network primary care clinics will be included. To participate, clinical practices must be willing to identify and refer potential English-speaking and Spanish-speaking participants with T1D or T2D to the research team at the University of Colorado for eligibility screening and informed consent.

### Sample and Recruitment

#### Provider and Staff

Practice providers and staff participants will be individuals (N=3 per clinic) who work at 1 of the 2 rural clinics where R-D1D will take place. A sample of providers and staff will be asked in person, by phone, or by email to participate in key informant interviews.

#### Patient and Care Partner

English-speaking and Spanish-speaking adults with T1D and T2D living in the proposed study region (N=64) and care partners in the intervention group (up to N=16) will be eligible for participation. We will use both community recruitment in venues identified during the BCT, as well as work with each of the 2 participating practices to help identify patients with diabetes who will then be randomized to either the intervention or attention control arm. They will be recruited using strategies recommended by the BCT participants, building upon successful recruitment strategies used in past projects. For instance, practices will distribute recruitment materials with messages created by the community-academic partnership through BCT and design elements selected by the community. This sample size is appropriate for iterating the intervention.

### Eligibility Criteria

#### Provider and Staff

Participants will be eligible if they work at the rural clinic where R-D1D will take place and if they were involved with the planning or coordination of R-D1D or were a provider who had a patient participate in the R-D1D intervention. Possible participants could include physician, nurse, medical assistant, front desk support, and clinic administrator. There are no exclusion criteria for providers and staff.

#### Patient Participant

Participants will be eligible for inclusion if they (1) have a known diagnosis of T1D or T2D with any A_1c_ level; (2) are ≥18 years old; (3) live in a rural eastern Colorado; (4) speak and understand English or Spanish; and (5) are willing to participate in a telehealth intervention at home through Health Insurance Portability and Accountability Act (HIPAA)-compliant Zoom. Participants will be excluded if they (1) are participating in another diabetes study; (2) have significant cognitive impairment; (3) are pregnant or planning to become pregnant in the next year (as diabetes management recommendations are different in pregnancy); (4) have life expectancy of <6 months; or (5) plan to move during the time of the study.

#### Care Partner

Care partner participants will be eligible if they are (1) identified by the patient participant who randomized to the R-D1D intervention group; (2) are 18 years or older; (3) speak and understand English or Spanish; and (4) are willing to participate in a telehealth intervention with the patient participant. Care partners will not be directly recruited.

### Group Randomization

R-D1D will be delivered to patients who receive diabetes care at 2 practices, once in English and once in Spanish at each practice. Thus, each site will have a total of N=32 persons with disability split into 4 groups (8 English control, 8 English R-D1D, 8 Spanish control, and 8 Spanish R-D1D). For each site and language, the participants will be randomized using alternating blocks of 2 and 4.

### Existing D1D Intervention

The intervention is the adapted R-D1D from the existing D1D program. D1D was designed for adults with T1D and T2D and is delivered using a hybrid model that includes in-person small-group and individual DSMES sessions. D1D is delivered by an interdisciplinary faculty specializing in diabetes care, including a nurse practitioner, certified diabetes care and education specialist, pharmacist, exercise specialist, ophthalmology technician, chef, licensed clinical social worker, endocrinologist, and obesity medicine physician. The topics included pathophysiology, complications, screenings, laboratories, medication options, weight management, the importance of exercise, healthy eating, troubleshooting glucose levels, diabetes technology, and healthy coping with diabetes. A healthy breakfast, interactive lunch with a chef demonstration, and snacks were also provided (Multimedia Appendix 1). An emphasis on participants providing education and support to others in the group sessions was encouraged. The participants received written and digital educational materials, recipes, a copy of the Calorie King Book, and resistance bands.

We anticipate R-D1D adaptations may focus on healthy eating based on food available in rural communities, stigma surrounding mental health in rural communities as it relates to diabetes management, and program structure and timing.

### Adapted R-D1D Intervention

R-D1D will be delivered by an interdisciplinary team of health care professionals specifically trained in the D1D program, including a nurse practitioner, physician, registered dietitian, certified diabetes care and education specialist, pharmacist, and a licensed clinical social worker. The R-D1D program will be delivered by telehealth by members of the original D1D team. An overview of the D1D curriculum that will be adapted is detailed in Multimedia Appendix 1.

Intervention group participants will meet with study staff to assess the participant’s technology literacy, test the participant’s internet connection and chosen electronic device, and provide instruction and demonstration for connecting to the videoconference platform. More than 1 meeting may be required. These meetings will be held via the same platform used for R-D1D intervention to allow the opportunity to troubleshoot directly with the participant.

### Control Group

The control group will receive a standard patient education handout that covers the ADCES7 self-care behaviors (healthy coping, healthy eating, being active, monitoring, taking medication, problem solving, and reducing risks) [24].

### Implementation Strategies

Implementation strategies refer to “methods or techniques used to enhance the adoption, implementation, and sustainability of a clinical program or practice” [25]. For this study, we will use several implementation strategies [26], including the following: (1) BCT to adapt and tailor D1D for the rural setting; and (2) technical assistance and workflow design. Grounded in the principles of community-based participatory research, BCT is a process used by partnerships of community members and academic researchers to translate medical information and guidelines into concepts and messages that partnerships believe will be relevant and actionable to local communities [27-30]. Partnerships develop materials and dissemination strategies that use and reflect local culture and assets to effectively move messages and materials to the desired audience. In the traditional BCT, community-academic partnerships are guided by the questions, “What is the message to our community?” and “How do we effectively share these messages with the community?” [31]. The process relies on the community members’ and researchers’ unique expertise, experiences, and perspectives. For this study, a modified BCT process will be used to adapt the existing D1D program for use in rural communities and primary care practices. Telehealth technical assistance will be provided by the community research liaisons or practice facilitators as needed.

### Outcomes and Measures

The outcomes for this study are informed by the Reach, Effectiveness, Adoption, Implementation and Maintenance (RE-AIM) framework [32]. We will use multiple methods (qualitative and quantitative) to assess select RE-AIM domains. Reach and Effectiveness are both patient-level outcomes. Reach refers to the number and percent of eligible patients who participate in the intervention, characteristics of those who participate and complete the measures, and reasons for not participating. Effectiveness refers to clinical and behavioral patient-level outcomes. Adoption, Implementation, and Maintenance are practice-level outcomes (maintenance can also refer to patient-level maintenance of health outcomes over time). Adoption refers to the number, percent, and characteristics of clinical practices who were invited to offer the intervention to their patients who participate in the study. Implementation refers to consistency of delivery of the intervention as intended (ie, fidelity), as well as practice perceptions of the acceptability, appropriateness, and feasibility of delivering the intervention in their clinical practice setting [33]. Maintenance refers to sustained effects on patient outcomes over time and to continued intervention delivery within the clinical setting beyond the study period, which will be explored in a future study.

#### Practice-Level Adoption, Implementation, and Maintenance

We will conduct baseline practice characteristics surveys, practice culture surveys, and individual semistructured interviews with 3 providers or staff at each practice (N=6) to assess practice characteristics and context, perceived value and fit with practice goals and priorities, feasibility (including resources required and perceived burden), and sustainability (including factors influencing interest in continuing to offer R-D1D when available). We will also administer valid, reliable surveys on perceived R-D1D intervention acceptability, feasibility, and appropriateness using the scales developed by Weiner et al [33]. Interviews and surveys will be conducted after each cohort is delivered.

#### Patient-Level Reach

We will capture data on R-D1D attendance, percent of R-D1D sessions attended, R-D1D program satisfaction, and patient reasons for participating and not participating.

#### Patient-Level Effectiveness

For both intervention and control patients, patient-reported outcome measures will include psychosocial (diabetes distress measured via Problem Areas in Diabetes scale [34,35] and Family and Friend Involvement in Adults’ Diabetes [36]), and behavioral (measured via Self-Care Inventory Revised [37]). Patient-level clinical outcome measures will include A_1c_, blood pressure, and BMI. A_1c_ will be obtained by home kit. These data will be collected at baseline and 3-month follow-up.

### Plans to Promote Participant Retention and Complete Follow-up

R-D1D is designed to be completed in 1 day. Study staff will encourage patient participants to participate in the study activities (intervention, follow-up surveys, and interviews) through phone calls, text messages, and email reminders.

### Data Management and Ethics Approval

Data use agreements among participating organizations have been established. The study procedures were approved by the University of Utah Multiple Institutional Review Board on October 28, 2020 (IRB_00133179).

### Statistical Methods and Analyses

#### Quantitative Analysis for RCT

Evaluating Reach will include description of the characteristics of those enrolled and screened ineligible and provide preliminary data on recruitment and retention capabilities for future studies. Quantitative metrics for recruitment and retention rates will inform population point estimates and 95% confidence intervals. For example, a 70% retention of 64 participants leads to a point estimate and CI of 70% (SD 6).

Descriptive statistics will be used to summarize R-D1D reach, adoption, and implementation at the participant and practice level. Special attention will be used to stratify outcome measures based on diabetes type, gender, and clinic site. For quantitative measures on feasibility, appropriateness, and acceptability, we will use the validated scales by Weiner et al [33]. Each of the 3 domains is composed of 4 Likert-scale questions (Completely Disagree 1 to Completely Agree 5). Our benchmarks for success will be to obtain domain means of 4 or greater.

#### Patient Level Effectiveness Analysis

Descriptive statistics will be used for intervention and control groups at baseline and 3 months stratified by language including outcomes from (1) surveys assessing diabetes distress as measured by the Problem Areas in Diabetes (PAID) scale and behavioral diabetes self-care as measured by the Self Care Inventory-Revised Version (SCI-R); and (2) clinical measures including A_1c_, blood pressure, and BMI. We will estimate population standard deviations so as to determine the sample size needed for a fully powered study to test for clinically meaningful improvements in outcomes [38,39]. Exploratory mixed-effects models using maximum likelihood estimation will be used to examine change between baseline and 3 months comparing intervention and control groups. This analysis is in keeping with an intent-to-treat standard and allows the use of all available data for analyses regardless of missing data. Combined, the results from this study will provide an initial indication of patient-level effectiveness as a clinically meaningful treatment effect in preparation for a larger effectiveness trial.

#### Missing Data

Missing Values Analysis tools will be used to describe if there are patterns to missing data. However, for the mixed models with random effects and maximum likelihood, it is not necessary to eliminate observations from participants who subsequently drop out, nor is it necessary to impute individual observations. To minimize loss of scale scores due to missing items in a computed scale such as PAID and SCI-R, we will prorate the respondents’ score based on their answered items. If a respondent is missing more than 30% of the items in a computed score, we will code the scale score as missing, thereby ensuring psychometric validity.

#### Qualitative Analysis

Qualitative descriptive analysis will be used to evaluate practice provider and staff interviews. Audio-recorded interviews with participants and practice providers or staff will be transcribed. Using standard qualitative content analysis, all investigators will review data, codes, and categories. Matrices will be used to organize codes by demographic variables (diabetes type, gender, and clinic site), patient reach, and practice-level adoption and implementation. Overarching themes will then be developed. Qualitative and quantitative data will then be triangulated to assess the R-D1D intervention.

#### Data Monitoring

The study team has developed data collection protocols to ensure maximum compliance and protection of informed consent documents, surveys, personal health information, and audio-recorded sessions (for interviews). The participants will complete electronic surveys via a weblink to REDCap (Research Electronic Data Capture), a HIPAA-compliant web-based application hosted at the University of Utah Center for Clinical and Translational Science, which will securely store and protect data. No names will appear on the surveys; however, the survey data will be linked to a practice location using an identification number. Survey data will be confidential and will only be used by the study team.

Interviews with practice staff will be conducted by the research team and will be recorded and transcribed with the respondent’s permission. No protected health information will be collected from the interviews, and all data will be de-identified. All audio files from the interviews will be stored on a secure server maintained by the University of Utah College of Nursing information technology team, and only the study team will have access to the data.

Data collected from the medical chart (ie, A_1c_, blood pressure, and BMI) or by phone (if participants are unable to complete electronic surveys) will be hand-entered by trained practice facilitators. Audio recordings of the participant interviews will be stored securely in a shared file system that only the study team will have access to. This file system will be backed up continuously and protected by the University of Utah firewalls.

A_1c_ data will be collected using home A_1c_ kits, which require 4 drops of blood (similar to checking a glucose level). The participants will mail the A_1c_ kits directly to the laboratory and will be tracked with a tracking number to ensure privacy and will allow the study team to link the data to the participant once the A_1c_ has been processed.

#### Harms

Risk of harm to participants (patient, practice, and staff) is minimal. Should any harm occur, it will be reported to all involved institutional review boards and the data safety monitoring team in accordance with institutional and federal policies.

## Results

We completed BCT [40] and have begun recruiting participants for the RCT component of the study. This study is expected to conclude in July 2022.

## Discussion

R-D1D will be developed in collaboration with patients with diabetes and other community members living in rural communities, providers and staff from participating practices, and academic clinician-researchers and their research teams.

While rural residents are burdened by higher rates of diabetes compared to their urban counterparts, it is difficult to access diabetes specialty care in rural areas. There is a critical need to test interventions to address diabetes health disparities in rural areas, in partnership with primary care practices, where the majority of people with diabetes receive their diabetes care in these areas. This study will adapt the D1D program, an effective, time-efficient diabetes self-management education and support program for adults with diabetes at University of Utah, for delivery in English and Spanish to rural residents of Eastern Colorado. Feasibility and acceptance will be evaluated. If successful, this project has potential for improving diabetes care nationally, not just for the residents of Colorado.

This project can provide valuable information on a program of research that is innovative and timely and has the potential to impact the delivery of diabetes specialty care in Spanish and English for rural regions beyond Colorado.

## Appendix

Multimedia Appendix 1 Comparison of the existing D1D (Diabetes One-Day) intervention and the adapted R-D1D (Rural Diabetes One-Day) intervention.

## Abbreviations

BCT: Boot Camp Translation
D1D: Diabetes One-Day
DSMES: diabetes self-management education and support
HIPAA: Health Insurance Portability and Accountability Act
PAID: Problem Areas in Diabetes
R-D1D: Rural Diabetes One-Day
RCT: randomized controlled trial
RE-AIM: Reach, Effectiveness, Adoption, Implementation, and Maintenance
REDCap: Research Electronic Data Capture
SCI-R: Self-Care Inventory-Revised Version
T1D: type 1 diabetes
T2D: type 2 diabetes
