# Supplementary figures and images for: A Telehealth Diabetes Intervention for Rural Populations: Protocol for a Randomized Controlled Trial
Source: JMIR Res Protoc. 2022 Jun 14;11(6):e34255. doi: 10.2196/34255 (PMC9240926; doi:10.2196/34255)

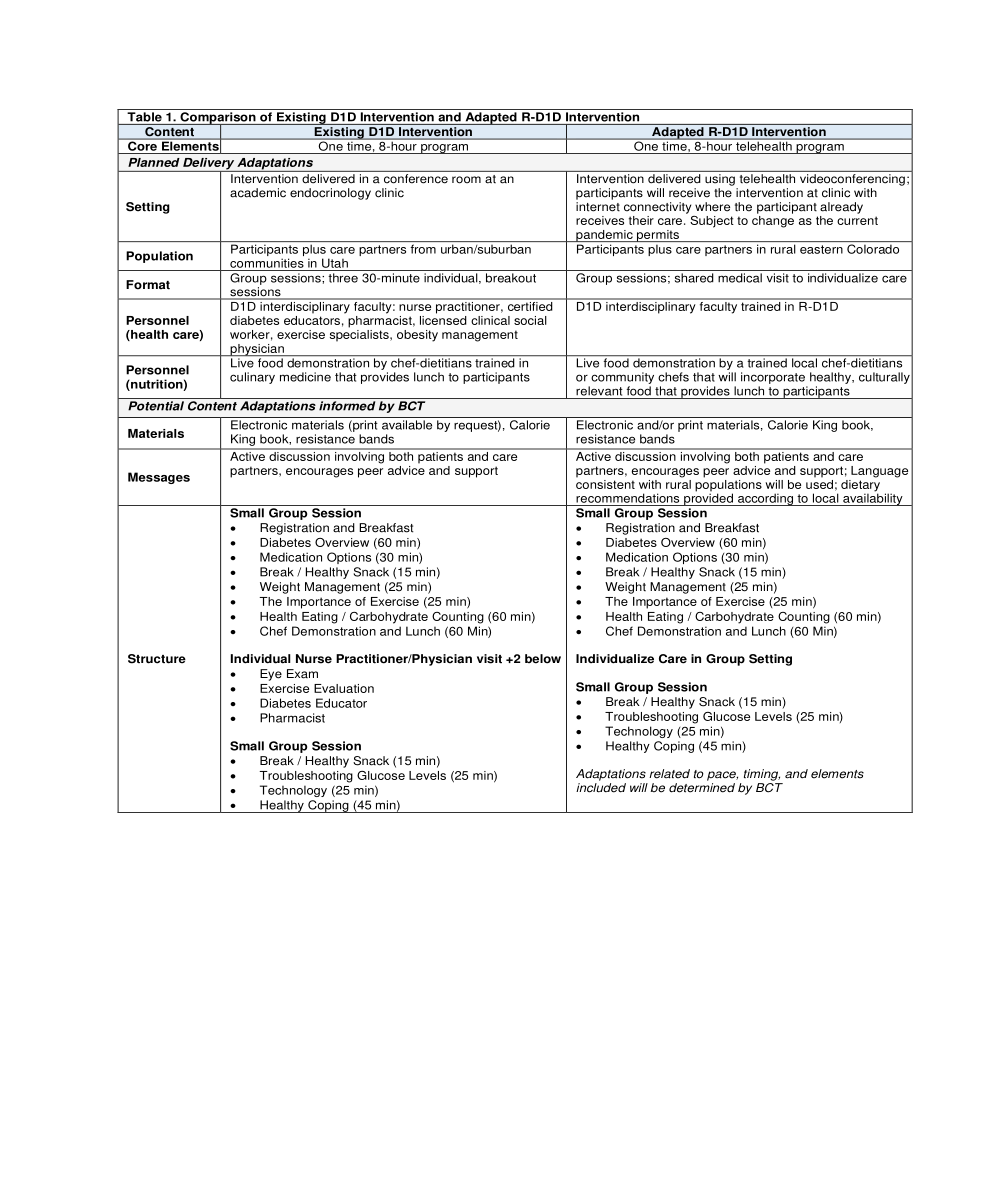

Supplement: Multimedia Appendix 1 [file resprot_v11i6e34255_app1.png]
